# Artificial Teeth Mounted on Plate, Adapted to the Mouth over Diseased Roots

**Published:** 1849-07

**Authors:** 


*Artificial Teeth Mounted on Plate, Adapted to the Mouth over Diseased Roots.*—The application of artificial teeth mounted on plate, over roots of natural teeth, whether in a healthy or diseased state, as practiced, by many calling themselves dentists, is productive of the worst and most injurious of consequences. Every practitioner of experience is aware of the deleterious effects that result from it, and yet there are many in the profession, so unscrupulous, as to be guilty of this species of malpractice almost daily. The roots of natural teeth when covered by a plate, at once become a source of irritation to the gums, causing them to inflame, become spongy, exhale a fetid odor, and often to suppurate. Teeth thus applied, however perfectly, in a mechanical point of view they are constructed, are never worn with comfort. In the majority of cases they are absolutely a constant source of annoyance to the patient, and were it not for false pride, they would not be tolerated.

Patients are not so much to blame, although the fear of pain may induce many to object to the removal of any roots of teeth, or dead or useless teeth which may be in their mouth at the time, as dentists. If, after stating the consequences that will inevitably result from their presence, the patient still persist in retaining them, it is the duty of the dentist to refuse his services. The practitioner should never sacrifice his better informed judgment to the wishes or whims of his patient. Whenever he can consent to do this, in a matter involving consequences so serious, he ceases to be worthy of confidence, and all claims to respect from his professional brethren. There was a time, when the means of retaining artificial teeth in the mouth were not as well understood as at present, when a dentist was justifiable in using the roots of one or more teeth for this purpose. But in the present advanced state of dental prosthesis, no such excuse can be plead in justification of the practice, and among conscientious scientific practitioners we believe it is never tolerated.

The foregoing remarks may, by some, be thought rather strong, but believing them, as we conscientiously do, to be true, we have made them at the suggestion of a professional friend, for the benefit of the young practitioner who may not have had an opportunity of observing the pernicious effects produced by the application of artificial teeth, under such circumstances. We have had frequent opportunities of noticing them, and have condemned the practice on more occasions than one.—*Balt. Ed.*

